# A narrative discussion on the impact of respiratory infectious diseases on dental education in China: implications for curriculum design and student outcomes

**DOI:** 10.3389/fdmed.2026.1897947

**Published:** 2026-09-04

**Authors:** Shuhao Xu, Xiaolong Li, Wei Li, Ruijie Huang

**Affiliations:** 1 Department of Stomatology, Deyang People’s Hospital, Deyang, China; 2 Department of Respiratory and Critical Care Medicine, Deyang People’s Hospital, Deyang, China; 3 Department of Pediatric Dentistry, State Key Laboratory of Oral Diseases & National Center for Stomatology & National Clinical Research Center for Oral Diseases, West China Hospital of Stomatology, Sichuan University, Chengdu, China

**Keywords:** aerosols, artificial intelligence, COVID-19, dental education, dental students, respiratory infectious diseases, virtual reality

## Abstract

**Objective:**

Recent outbreaks of respiratory infectious diseases, transmitted via aerosols, have placed dental healthcare workers at high risk. Traditional dental education, based on student–teacher–patient interactions, faces unprecedented challenges across all stages. From admissions to classroom teaching, laboratory instruction, clinical training, and final examinations, every aspect of dental education is undergoing significant challenges and is in urgent need of change.

**Methods:**

This narrative review with content analysis synthesized literature on the impact of respiratory infections on dental education. We systematically searched PubMed, Web of Science, and CNKI (2020–2026) using relevant keywords. Two reviewers independently screened and selected studies. Data were thematically analyzed into five core domains: classroom teaching, laboratory teaching, clinical teaching, student admissions/assessment, and licensing examinations.

**Results:**

In classroom teaching, online platforms and AI-augmented PBL/CBL were rapidly adopted, yet persistent issues included network instability, limited interaction, digital inequities, and heavy reliance on student self-discipline. In laboratory teaching, AR/VR and iLab-X enabled remote training, but were constrained by high costs, poor haptic realism, inability to replicate patient emotions, and equipment dependence. In clinical teaching, enhanced infection controls—including PPE, rubber dams, and HEPA filtration—were implemented, though bio-aerosol risks, reduced patient flow, and limited clinical exposure remained. In admissions, online exams with dual-camera proctoring, open-ended questions, AI-generated items, and virtual OSCEs were adopted, while challenges persisted in cheating prevention, subjective scoring, validity, and privacy. Licensing examinations remained in-person with strict protection; VSP showed promise but lacked robust evidence and incurred high costs. Overall, technological innovations enabled educational continuity, yet significant infrastructure gaps, inequities, and unresolved validity issues persist across all domains.

**Conclusion:**

China's dental education rapidly adapted to respiratory epidemics through online teaching, simulation, and enhanced infection control, yet persistent challenges remain in digital equity, cost, validity, and infrastructure. These innovations are important primarily because they directly influence student learning outcomes—including knowledge acquisition, clinical reasoning, and skill retention—while also shaping curriculum design. Future preparedness requires sustained investment, faculty development, equitable access, and rigorous longitudinal research to validate skill transfer to clinical practice, ensuring that technological advances ultimately translate into measurable improvements in student competency and patient safety.

## 1 Introduction

In recent years, emerging respiratory infectious diseases have continuously appeared (1), ranging from severe acute respiratory syndrome (SARS) in 2003, influenza A (H1N1) in 2009, and Middle East respiratory syndrome (MERS) in 2012, to coronavirus disease 2019 (COVID-19). Each epidemic and outbreak of respiratory infectious diseases has had an impact on the field of dental education. This is attributable to the fact that various respiratory viruses are primarily transmitted via droplets and aerosols between individuals; dental healthcare workers, in particular, are regarded as one of the populations at greatest risk of infection, owing to their sustained exposure to aerosol-producing procedures (2, 3). Dental professionals themselves also serve as vectors for the transmission of respiratory infectious diseases (4) and may cause outbreaks of nosocomial infections (5). In general, dental medicine is a challenging discipline that places strict demands on the comprehensive qualities of students, beginning with theory and culminating in practice. Although in recent years, with the gradual popularization of online education and the application of teaching tools such as manikin heads and virtual simulation systems, dental education has continuously introduced innovations in teaching methods. However, particularly in the wake of various respiratory infectious disease pandemics especially COVID-19, dental education continues to face numerous deficiencies in areas such as classroom instruction, laboratory training, clinical teaching, student admissions, and dental licensure examinations. Because dental medicine is more complex than other disciplines, dental students not only need to master a large amount of theoretical knowledge but also need to complete more diagnostic and treatment procedures under the guidance of clinical instructors to improve their clinical skills (6). Moreover, dental clinical procedures—whether pulp opening, tooth preparation, or periodontal scaling and root planing—inevitably generate aerosols (7). Furthermore, traditional dental education is based on a triadic relationship involving students, teachers, and patients. However, in the face of epidemics and outbreaks of respiratory infectious diseases, people need to maintain social distancing as much as possible and reduce face-to-face interactions (8). In this context, the difficulties faced by dental education become particularly prominent (9). Numerous studies have documented that dental students experience stress and other mental health consequences, including anxiety and depression. These pressures were potentially intensified during the pandemic, thereby further undermining the effectiveness of didactic instruction. Additionally, the transition from face to face to online learning has posed challenges for students in adapting to novel technologies and clinical practices, which may contribute to heightened levels of stress and anxiety (10, 11). Thus, the field of dental education urgently requires a comprehensive set of countermeasures to combat potential future epidemics and outbreaks of various respiratory infectious diseases.

Difficulties, however, often serve as drivers of change—they are not only challenges but also new opportunities. Despite the extensive body of research reporting on dental education reforms during the COVID-19 pandemic, the scope of dental education remains broad, and a comprehensive set of response measures covering the full continuum of dental education—encompassing classroom teaching, laboratory teaching, clinical teaching, student admissions and assessment, and licensing examinations—is still lacking. Dental education faces a critical gap in preparedness across all domains, and understanding this impact is essential for guiding future curriculum reforms and safeguarding student competency. In summary, this article discussed the influence of various respiratory infectious disease epidemics on essential components of dental education and puts forward practical solutions, aiming to equip dental educators in China with new approaches to tackle the difficulties presented by respiratory disease epidemics or outbreaks.

## 2 Methods

This narrative review was conducted to synthesize the existing literature on the impact of respiratory infectious diseases on dental education. The methodological framework was informed by established guidance for narrative reviews in health professions education (12, 13). A systematic literature search was performed in three electronic databases: PubMed, Web of Science, and CNKI (China National Knowledge Infrastructure), covering the period from January 2020 to June 2026. The primary search strategy combined keywords related to respiratory infections (e.g., “respiratory tract infections,” “COVID-19,” “influenza,” “airborne transmission”) with terms related to dental training (e.g., “dental education,” “dental curriculum,” “clinical teaching,” “simulation training”) using Boolean operators (AND/OR). Additionally, the reference lists of retrieved articles were manually screened to identify further relevant studies.

Two reviewers independently performed title/abstract screening and then full + text assessment against predefined eligibility criteria. Discrepancies were resolved through discussion. The researchers included peer-reviewed original research articles and systematic reviews published in English or Chinese that directly addressed the challenges, adaptations, or pedagogical responses of dental schools during outbreaks of respiratory diseases. Conference abstracts, editorials, opinion pieces, and publications in languages other than English or Chinese were excluded.

Data were synthesised thematically, organising the findings into five core domains derived inductively from the literature: (1) classroom teaching, (2) laboratory teaching, (3) clinical teaching, (4) student admissions and assessment, and (5) licensing examinations. No meta-analysis was performed given the heterogeneity in study designs, outcomes, and educational contexts. The narrative synthesis allowed us to integrate diverse evidence and provide a broad overview of the field, while explicitly acknowledging the limitations inherent in such a synthesis.

## 3 Results

### 3.1 Classroom teaching

In China, although dental medicine and clinical medicine are both classified as first-level disciplines, the unique nature of dental medicine places a stronger emphasis on practical training compared to other medical disciplines. Therefore, within the original educational system, the main focus has been on knowledge of dental specialties and clinical skills training, with relatively shallow coverage of public health disciplines and clinical medicine content. As a result, during past outbreaks of respiratory infectious diseases, the lack of relevant knowledge reserves and basic protective skills was one of the prominent issues faced by dental education (14). Therefore, consideration should be given to adding courses in dental public health to classroom teaching and integrating relevant content of infectious diseases into the dental medicine curriculum, so as to help students develop good awareness of prevention and control, and to better respond to epidemics and outbreaks of respiratory infectious diseases (15, 16).

During epidemics and outbreaks of respiratory infectious diseases—especially the COVID-19 pandemic—dental schools modified their curricula in order to uphold social distancing and minimize the risk of viral transmission (17). Most institutions suspended traditional offline classroom teaching and replaced it with modern online classroom teaching, including live or pre-recorded online lectures, online seminars, remote case discussions, and so on (18, 19). In the context of the “Internet+” and the 5G era, dental students can participate in online classroom teaching activities from safe locations. In recent years, various teaching models such as Massive Open Online Courses (MOOCs), Small Private Online Courses (SPOC), and flipped classrooms have emerged, offering diverse formats and gradually becoming auxiliary tools for traditional classroom teaching in universities (20). The rapid integration of digital technologies in dental education has given rise to new teaching models, such as MOOCs, which offer standardized, scalable, and flexible learning opportunities. Evidence suggests that in health professions education, MOOCs and structured online modules have been shown to improve knowledge retention, clinical reasoning, and learner satisfaction (21). SPOC differ from MOOCs and other open online courses. Although their teaching resources are similar to those of MOOCs, SPOC can be customized to meet participants' needs due to their diverse instructional formats, efficient dissemination, and high precision of course content, thus placing greater emphasis on personalized learning (22). In the typical flipped classroom model, assignments that are conventionally completed as homework are instead performed in class with teacher interaction, while listening to lectures or viewing course-related videos is undertaken at home. The essence of the flipped classroom lies in the reversal, or “flipping,” of activities traditionally associated with class time and self-study time (23). These new teaching models not only serve as a valuable supplement to traditional instructional approaches but can also be readily transitioned to online formats, thereby helping to avoid large gatherings and reduce the risk of disease transmission. Although online teaching offers many advantages, such as abundant teaching resources, the ability to transcend time and space limitations, rapid replication and dissemination, and flexible and diverse interactions (24), achieving a high degree of sharing of teaching resources to some extent, the sudden outbreak of respiratory infectious diseases forced an abrupt transition from offline to online teaching models, which inevitably led to certain disruptions. These include unstable online classroom signals due to limitations in hardware and software conditions as well as differences in network quality, difficulty for teachers to gauge students' engagement, restricted teacher-student interaction, and an insufficient learning atmosphere (25). The reason for this may be that dental education has never attempted to use online classroom teaching as a mainstream instructional model, so teachers, students, and various teaching units are unable to adapt well to this sudden transition. Moreover, the teaching content of dental medicine is extensive and involves a great deal of fundamental and practical knowledge, making it difficult to make the otherwise dry classroom knowledge delivery more engaging (26). At the same time, the quality of online classroom teaching is highly dependent on students' self-discipline and network equipment, which presents a brand new challenge for the vast number of teachers, students, and dental education institutions. The gap between students who have access to IT technology and those living in rural areas without adequate technological facilities is also one of the major concerns (27). Indeed, access to online teaching and educational resources is highly unequal: many students from low socioeconomic backgrounds face more severe challenges than their peers from higher socioeconomic backgrounds and may perform below normal levels (28). Therefore, compared with traditional classroom teaching models, the establishment and formation of online classroom teaching's philosophy, content, methods, resources, and objectives will all be disruptive innovations and reforms carried out on the basis of traditional teaching (29).

In online classroom teaching, one of the most important elements is the inclusion of real-time online interaction between teachers and students, along with encouraging students to engage in self-directed learning. While providing students with online courses and remote resources, even more emphasis should be placed on guiding them on how to learn independently. Therefore, how to capture students' interest in learning and stimulate their motivation to learn becomes the key to online classroom teaching. Online classroom teaching should not be a “replicated” face-to-face video teaching broadcast on the internet; instead, it should encourage students to use various information tools and online resources, extensively consult materials, and engage in two-way interaction with teachers, so as to broaden students' horizons (25). Finally, efforts should also be made to train and enhance teachers' information-based teaching capabilities, guiding the transformation of teachers' roles from users of teaching resources to developers and designers, carefully designing online courses rather than simply replicating offline classrooms, and incorporating such information-based teaching capabilities into assessment indicators (30).

The rapid advances in artificial intelligence (AI) technology have sparked a revolution in medical education, and these technologies may offer promising solutions to address these challenges. Large language models (LLMs), such as ChatGPT (OpenAI), which are capable of automatically processing and generating natural language, have been integrated into classroom teaching motheds like Problem-Based Learning (PBL) and CBL (Case-Based Learning), enriching the learning experience (31). AI can further assist PBL and CBL by leveraging generative models such as LLMs, generative adversarial networks (GANs), variational autoencoders (VAEs), and diffusion models, to create synthetic multimodal cases that integrate medical texts, images, and physiological signals, thereby simulating real clinical scenarios (32). The integration of novel AI technologies into PBL and CBL is expected to enhance students' knowledge acquisition and satisfaction (31). Meanwhile, generative models enables personalized educational experiences by tailoring content to individual student needs and providing targeted feedback. These tools can simulate complex diagnostic scenarios (33), allowing students to refine their clinical decision-making in a safe environment. Nevertheless, the implementation of AI in dental education continues to face barriers, including resistance to change, knowledge gaps, fear of misuse, cost, accessibility, effectiveness, reliability, ways of thinking, ethical concerns, as well as risks like plagiarism, bias, or a lack of transparency (34).

### 3.2 Laboratory teaching

Fine and flexible clinical manipulation is an essential skill for dentists, and practical education, including laboratory teaching, is an effective method for improving dental students' clinical operational skills. Laboratory teaching also serves as an important transitional phase for dental students from the pre-clinical stage to the clinical internship stage. In recent years, although the forms and platforms of online theoretical teaching have continuously evolved and upgraded, laboratory teaching has remained a bottleneck limiting program establishment and the improvement of teaching quality. During epidemics and outbreaks of respiratory infectious diseases, classroom teaching can be relatively easily converted to an online model, whereas laboratory teaching is more difficult to transition; therefore, curriculum reform in the latter faces greater challenges (2). For certain experimental content that can be performed independently, such as using wax to build tooth models or bending orthodontic archwires, a distance teaching model can be adopted: the teacher first demonstrates the procedures, and then students complete the experimental tasks on their own. In the past, teaching often relied on extracted natural teeth brought by the students themselves; however, the 2003 Guidelines for Infection Control in Dental Health-Care Settings issued by the U.S. Centers for Disease Control and Prevention (CDC) clearly stipulate that extracted natural teeth must be sterilized before being used in dental laboratory teaching and scientific research (35, 36). Both high-temperature high-pressure steam sterilization and hydrogen peroxide low-temperature plasma sterilization techniques can be used for sterilizing extracted teeth for educational purposes. Therefore, extracted natural teeth can be safely used in laboratory teaching. However, extracted teeth intended for bonding strength experimental research should not be sterilized using hydrogen peroxide low-temperature plasma sterilization (37). In addition, artificial plastic teeth have also been adopted in laboratory teaching. Nevertheless, the material of plastic teeth differs significantly from that of natural teeth, making it difficult to simulate actual intraoral operating conditions. Moreover, for the majority of procedures that rely on specialized teaching equipment such as manikin heads and handpieces, online teaching models are not feasible, and only offline teaching methods can be adopted.

With the convergence of augmented reality (AR) and virtual reality (VR), the landscape of dental education and clinical practice is undergoing profound transformation. These innovative technologies are ushering in a new era of medical training, and are increasingly recognized for their potential to enhance learning and clinical outcomes (38). In dental schools with adequate resources, AR and VR technology can be used for experimental skills training (39). AR and VR allow dental students to practice and improve their skills in a safe, controlled environment (40) without compromising patient health, while also protecting them from the threat of respiratory infectious diseases. Three-dimensional virtual simulation interactive software makes teaching models repeatable and reversible, better simulates real conditions, and enables precise evaluation and feedback on training outcomes, thereby playing a positive role in improving the quality of dental education (41). Virtual simulation systems can be used for training in clinical skills such as oral examination, local anesthesia, periodontal examination and scaling/root planing, treatment of dental diseases, and implant placement (42–44). As material loss is no longer a factor when students practice fine motor skills, VR haptic simulation reduces students' anxiety about fear of failure. With VR haptic training, teachers are free to focus on those students who need more help, enabling personalised learning. As mentioned above, VR haptic technology facilitates skill acquisition and enhances student confidence (45). The Simodont system, jointly developed by the Academic Centre for Dentistry Amsterdam (ACTA) and Moog, utilizes VR technology combined with teaching courseware to simulate various oral problems of patients, thereby enhancing students' interest and motivation during hands-on training and enabling a better integration of theoretical knowledge (46). In China, the rapid development of VR technology has likewise driven its application in dental practical education. For example, the UniDental (formerly iDental) system developed by Beihang University can be used for laboratory teaching in tooth cutting, periodontal examination and treatment, and other areas (47). The Peking University School of Stomatology has applied a VR-based periodontal operation experimental teaching system for the training and assessment of periodontal skills. The College of Stomatology of Chongqing Medical University has used VR technology to design experimental teaching, moving the dental technology laboratory simulation online, and has also established a virtual simulation system for tooth extraction procedures. West China School of Stomatology, Sichuan University, in collaboration with Beihang University and Beijing Zhonghui Virtual Reality Research Institute Co., Ltd., has developed China's first vision-haptic fusion multifunctional dental implant surgery simulator, among others (48). However, due to the high cost of virtual simulation systems, dental schools generally purchase only a small number of them. If the number of students is too large, it may fail to meet weekly teaching demands. VR devices must provide high-resolution graphics and extremely low latency in order to effectively simulate surgical procedures. Augmented reality, on the other hand, must seamlessly integrate digital information with the real-world environment, ensuring precise alignment without causing disorientation (49). Additionally, current VR technology still has shortcomings, including the realism of force feedback and the authenticity of the user interface (50). These simulation systems are often incapable of replicating patients' emotional or physical responses—such as anxiety or fear—which are crucial factors in shaping both treatment strategies and clinical outcomes (51). Moreover, the mainstream VR systems currently used in dental schools still require specialized equipment, making it impossible for students to complete their learning at home.

In China, to continuously monitor the external connectivity and service status of national virtual simulation experimental teaching projects, promote open sharing, and enhance application effectiveness and outcomes, the Ministry of Education commissioned the Higher Education Press to establish the National Virtual Simulation Experimental Teaching Project Sharing Platform, named “iLab-X” (http://www.ilab-x.com). Many projects on the iLab-X platform have been used in online-offline integrated experimental teaching at regular universities, and these educational resources are accessible to students on personal computers, demonstrating unique value especially during epidemics and outbreaks of respiratory infectious diseases (52). However, there is still considerable room for improvement in terms of simulation realism, experimental methods, interaction design, data collection and aggregate analysis, system usability, and teaching team support for virtual simulation experimental teaching projects at present (53).

### 3.3 Clinical teaching

An aerosol is defined as a dispersion system in which liquid or solid particles generated by humans, animals, instruments, or equipment are suspended in a gaseous medium. A bio-aerosol refers to an aerosol containing any one or more types of microorganisms (54). Bio-aerosols can remain suspended in the air for several hours, travel long distances with airflow currents, and eventually settle on and contaminate surfaces. Depending on the particle size of the aerosol, they can penetrate different parts of the respiratory tract, even reaching the alveoli, thus forming a potential route of infection for pathogenic microorganisms (17). Many dental procedures generate aerosols, such as the use of handpieces, three-way syringes, and ultrasonic scalers (55, 56). Studies have shown that respiratory viruses have three main transmission routes in dental diagnosis and treatment: transmission through inhalation of cough droplets, transmission through the oral, nasal, or ocular mucosa, and direct contact transmission (18). Many dental schools have adopted the measures recommended by CDC and the American Dental Association (ADA) to reduce the risk of transmission (57). In clinical teaching, to control virus transmission, most affiliated hospitals of dental schools in China did not allow dental students to enter treatment areas during the most severe outbreaks of respiratory infectious diseases. On one hand, this was to reduce the number of people moving within the clinical settings; on the other hand, it was to protect student safety. When the epidemic became relatively stable, a strategy of ‘spaced-out dental chairs’ was adopted, and students were required to wear protective clothing during clinical sessions. In addition, the routine use of rubber dams can also reduce the spread of dental bio-aerosols (58). Therefore, during epidemics and outbreaks of respiratory infectious diseases, the frequency of rubber dam use increased significantly compared with before. However, because undergraduate interns perform clinical operations more slowly than standardized trainees or visiting trainees (59), clinical teaching schedules were reduced, and the implementation of clinical teaching activities became restricted. During respiratory infectious disease outbreaks, the high risk of transmission has led most dental institutions/clinics to close, limiting their services to emergency dental treatment only (60). Reduced patient exposure may also translate into inadequate clinical skills training among dental students (61).

The epidemics and outbreaks of various respiratory infectious diseases have elevated the infection control requirements for clinical teaching to an unprecedented level. Factors such as environmental cleaning, hand hygiene, and personal protective equipment are crucial for preventing and controlling respiratory infections and are also the main focus of current research in this area (62, 63). During outbreaks of respiratory infectious diseases, in addition to the control and reduction of bio-aerosols mentioned above, teaching units should also require students to strictly adhere to infection control measures, maintain necessary social distancing, and reduce the number of people in the clinical teaching space (64). During clinical teaching, the three-level pre-examination triage system should be strictly implemented, patient flow should be reasonably arranged to avoid crowding and maintain social distancing, and effective ventilation and environmental disinfection measures should be ensured (65, 66). During teaching and clinical procedures, both teachers and students should strictly adhere to standard precautions, which assume that every healthcare worker and patient is a potential (low-risk) source of infection. Infection control measures should be designed on this basis and strictly implemented in daily work to minimize the risk of nosocomial infection through any route of transmission (54). For patients who are suspected or confirmed to be infected, or are close contacts, treatment should be postponed, while ensuring patient safety, until after the infectious period or the end of self-isolation (67). Meanwhile, strengthening the use of personal protective equipment (PPE), such as masks, gloves, face shields, goggles, and protective gowns, is a critical step in protecting patients and healthcare workers from respiratory infectious diseases, as previous studies have shown that appropriate use of PPE can reduce the risk of COVID-19 transmission (68). Encourage the use of hand instruments and minimally invasive dental techniques whenever possible, and also emphasize the use of rubber dams and high-volume suction to reduce aerosol generation (69). If dental treatment is necessary, it should be strictly carried out according to the three-level protection standards. In addition to further strengthening inter-appointment disinfection, patients should rinse with mouthwash for one minute before treatment to disinfect the oral cavity. Recommended mouthwashes include 1% hydrogen peroxide, 0.2% povidone-iodine solution, or 0.2% chlorhexidine rinse (7). Negative-pressure rooms can also be used to reduce the risk of aerosol infection, though such rooms are not widely available in dental clinics. Additionally, the U.S. CDC recommends air filtration systems with high-efficiency particulate air (HEPA) filters, as these devices can remove up to 99% of airborne pathogens within 23 min, thereby shortening room turnover time (70, 71).

### 3.4 Student admissions and assessment

The levels of dental students are quite diverse. In China, conventional full-time student populations include undergraduate students, master's degree students, and doctoral degree students. In addition, standardized residency trainees, specialty fellowship trainees, and visiting trainees are also important components of dental students. For admissions of the above six categories of students, except for undergraduates who are directly selected through the national college entrance examination into dental schools, most are required to take written examinations and interviews organized by dental schools. The written examination is generally administered by dental schools as a unified on-site test, while the interview stage selects outstanding students based on their written examination scores and conducts unified on-site interviews. During the COVID-19 pandemic, traditional on-site assessment methods posed significant infection risks. The challenge we faced was how to convert on-site activities to online formats while maintaining the same level of examination fairness as before. During outbreaks of respiratory infectious diseases, relevant policies require balancing student admissions and selection while protecting individuals and reducing pathogen transmission as much as possible (72). Therefore, online admissions are often the preferred form during epidemics and outbreaks of respiratory infectious diseases.

There are two main solutions for online admissions of dental students. The first is to conduct online written examinations and interviews under dual-camera monitoring (73). The second is to modify the exam content, replacing traditional standard-answer questions with open-ended questions that students must complete within a specified time. The first solution has become feasible with the support of various online conferencing software available today (62). For example, using a dual-camera setup for examinees—one camera positioned in front and another at the rear side—can simultaneously monitor students during written exams and interviews, making remote testing feasible. However, this solution still has problems such as high dependence on network and information equipment, lack of remote network technical measures to prevent cheating, and the inability to conduct practical skills assessments (74). The second solution replaces the exam content. Its advantage lies in the ability to more comprehensively evaluate students' knowledge levels and to some extent prevent cheating methods such as mutual copying. However, because open-ended questions involve greater subjectivity in both answering and grading, the validity of assessing student abilities is far from that of traditional models. Therefore, a more appropriate set of evaluation criteria needs to be established. From the students' perspective, this shift brought both opportunities and challenges. On the positive side, online admissions reduced travel costs, accommodation expenses, and time away from study, making the process more accessible for many (75). However, At the same time, a considerable number of students have been adversely impacted by factors such as insufficient work experience, internships, and clinical exposure in dentistry; disadvantageous course delivery and assessment policies; the loss of opportunities for dental school visits; and the objective social and personal hardships linked to the respiratory pandemic (76). Although evidence remains limited regarding whether online admissions assessments are comparable to on-site ones (77), but practice in China has shown that online re-examinations can produce the same screening effects as on-site re-examinations in the process of admitting medical postgraduates (75).

The COVID-19 pandemic has prompted changes in dental education, particularly in student admissions and assessment methods (78). In recent years, the combination of VR technology and AI has been gradually applied in student admissions and assessment, and approaches for evaluating candidates' various skills will continue to be explored. The advent of these novel technologies may offer safer and more equitable solutions for online student admissions and assessment in the event of future respiratory infectious disease epidemics. Objective Structured Clinical Examinations (OSCEs) have consistently served as a cornerstone of summative assessment in both undergraduate and graduate medical education. Several studies have reported that online virtual OSCEs are highly satisfactory and feasible in a range of disciplines, such as emergency medicine, general surgery, vascular surgery, and radiation oncology. Students gave generally positive feedback, appreciating the flexibility and safety of virtual OSCEs, and many emphasized the value of becoming familiar with telemedicine—a skill increasingly relevant in modern healthcare and aligned with global trends in medical education (79). Nevertheless, concerns persist regarding whether virtual OSCEs are truly comparable or equally acceptable, and a number of individuals have voiced reservations about the reliability of virtual assessment formats (80). AI may offer new possibilities for admissions and student assessment (81). AI is regarded favourably by students, owing to its capacity to deliver personalised learning experiences through progress tracking and immediate feedback, which in turn reinforces their comprehension of challenging topics. Consequently, a recent survey indicated that medical students demonstrated a desire to develop skills pertinent to the use of AI in the medical field during their undergraduate education (82). For example, in Objective Structured Practical Examinations (OSPEs), AI-generated examinations have demonstrated technical accuracy and effectively addressed core subtopics that are appropriate to learners' level (83). Additionally, AI can be applied to admissions and assessment processes for the purposes of generating test items, scoring examinations, and delivering feedback, thus substantially lessening the burden on educators while providing students with individualized formative feedback (84). Concerns persist with respect to the accuracy, potential bias, and ethical implications of AI in admissions and practical skills assessment (85). Although AI can ensure that students are assessed safely during outbreaks of respiratory infectious diseases, and has the potential to enhance current assessments by providing immediate feedback, personalised learning experiences, improving objectivity and consistency, and reducing educators’ workload, however, students also worry about algorithmic bias, lack of transparency in scoring, and the ethical implications of AI decisions affecting their academic future (85). VR and AR technologies have achieved certain successes in the field of dental education, particularly in laboratory teaching. Students appreciate the opportunity to practice in a safe, repeatable environment, which reduces their anxiety about making mistakes. Computerized objective assessment systems allow students to receive immediate, detailed digital reports on their performance, helping them understand their strengths and weaknesses (86).

### 3.5 Licensing examinations

Licensing examinations are intended to ensure that dental schools graduate competent dentists. Yet during the COVID-19 pandemic, numerous countries either postponed or partially suspended these assessments (87). Consequently, concerns have arisen regarding the potential implications for patient safety when such examinations are circumvented. Hence, the safe conduct of licensing examinations amid respiratory infectious disease outbreaks remains a challenge that must be confronted. For dental students, these examinations represent a critical milestone that determines their entry into professional practice. Licensing examinations in China mainly consist of the Practicing Physician Examination, the standardized residency training examination, and the specialty fellowship training examination. These are all national-level examinations, comprising two major components: a written test and a clinical skills test. The written portion can be administered at multiple different test sites within the same province. However, due to the constraints of clinical instruments and materials, the number of test sites for the clinical skills examination is relatively limited within each province, meaning that candidates may need to leave their place of residence to take the examination. From the students' perspective, this geographic limitation poses a major burden: they must often travel long distances, incurring additional costs and time away from their studies, while also increasing their exposure to infection during outbreaks. For written examinations, unlike student admissions, a large number of candidates take the exam during the same time period. Moreover, as these are national-level examinations, their format, content, and validity should not be easily altered. Therefore, neither a dual-camera proctoring model nor changes to the exam content can be adopted. Since the written exams can be administered at major test centers in nearby cities, candidates generally do not need to travel long distances. Thus, the most common approach is to arrange centralized in-person examinations at a suitable time when the pandemic has been brought under control, in compliance with rigorous prevention protocols (88). According to experiential reports, candidates were placed in separate rooms for the written examinations while adhering to social distancing protocols; the oral interviews were held in four distinct rooms within the examination facility, employing videoconferencing software and uniform laptops, thus guaranteeing that all candidates shared the same technical conditions. All examiners were located in their private offices at their respective hospitals. Thus, the candidates and the two examiners were separated from one another (87). For the clinical skills examination, due to multiple requirements such as appropriate venues and proctors, no satisfactory alternative has yet been found.

As an important tool for dental education reform, AI has been supported by recent studies showing that LLMs can pass medical licensing examinations, such as the United States Medical Licensing Examination (USMLE), and also demonstrate remarkable performance on US dental licensing examinations (89). However, current research on the application of LLMs in licensing examinations is still mainly focused on question generation (90), with a lack of studies on their use as scoring tools for licensing examinations, which may represent a future research direction. Thus, students remain cautious about relying on AI for evaluation, fearing that opaque algorithms might produce biased results. With the introduction of virtual reality technology, Virtual Standardized Patients (VSP) have emerged. These systems emphasize high interactivity, possess artificial intelligence and natural language understanding capabilities, and can consistently and realistically simulate specific clinical presentations as standardized patients (91, 92). The Nanjing Stomatological Hospital, Affiliated Hospital of Medical School, Nanjing University, was the first to apply VSP in the clinical skills examination of standardized residency training (dentistry) in 2020. The successful application of VSP demonstrates its considerable potential for use in licensing examinations. It can be used to assess medical students’ clinical examination and critical thinking abilities, and holds promise for providing new directions for reforming various aspects of licensing examinations, including clinical skills assessments (93). Most importantly, students emphasized that they want assessment methods that are not only safe and convenient but also fair, transparent, and capable of accurately measuring their readiness to care for patients.

## 4 Discussion

The majority of the evidence cited in this review was drawn from the lessons of the COVID-19, with the intention of generalizing these insights to the broader context of respiratory infectious diseases and their implications for dental education. This was attributable to the fact that it was the COVID-19 outbreak that first elevated the pre-existing deficiencies in dental education under epidemic conditions to an unprecedented level of scrutiny (94); furthermore, the substantial body of research generated during this period has offered valuable guidance. With respect to future recurrences of respiratory infectious disease epidemics, no comprehensive set of measures addressing the full continuum of dental education—encompassing classroom instruction, laboratory training, clinical education, student admissions and assessment, and licensing examinations—has yet been established. This constitutes the primary focus of the present review.

With regard to classroom instruction, the pandemic has compelled significant transformations in medical education globally—beyond stressing the need to integrate content on the prevention and management of respiratory infectious diseases into curricula, the principal change has been the transition from face-to-face to online delivery; some of these shifts have turned out to be more effective than pre-pandemic approaches. Virtual classrooms have afforded students greater flexibility, less commuting, and the opportunity to learn at their own pace (95). Meanwhile, the rapid development of AI technology has also opened up more possibilities for online virtual classrooms (31). Nevertheless, researchers continue to critically examine concerns related to the effectiveness of online instruction, its transferability to patient care, skill retention, scalability, cost, infrastructure, accessibility, and educational equity.

In the field of laboratory teaching, in addition to strengthening infection prevention and control measures in traditional laboratory settings, AR and VR technologies have also offered new approaches. These novel technologies not only enable students to learn through repeated practice without endangering patients, but are also capable of faithfully replicating the tactile sensations that practitioners may experience when handling actual specimens (96, 97), while simultaneously guaranteeing that students can perform their laboratory training in a secure setting, free from exposure to patients or their immediate surroundings. Nevertheless, these technologies also present several challenges, including high upfront costs, requirements for sustained technical infrastructure, faculty development needs, and the demand for additional longitudinal research to validate the translation of acquired skills into clinical practice, among other considerations (98).

In terms of student admissions and assessment, online platforms can ensure that these processes are conducted safely during outbreaks of respiratory infectious diseases (73). At the same time, AI assistance has also brought new vitality to OSCEs (83). While online interviews and assessments might be as effective as offline formats, persistent challenges remain, including the prevention of cheating and the current shortage of robust solutions for the virtual evaluation of clinical and laboratory skills (77).

Lastly, concerning licensing examinations, which encompass both written and clinical components, their status as national assessments means that their structure, content, and validity ought not to be changed without due consideration. Currently, other than choosing a relatively low-risk time frame during the outbreak and reinforcing infection control protocols, no superior alternative has been identified. Although VSP has already been used in practical assessments, there are still issues such as high economic costs, and insufficient evidence regarding the effectiveness of training and assessment in certain clinical skills (99).

Technology has changed the way we learn and access knowledge, especially with the introduction of digital devices and the internet. We are no longer limited by time or space, and information is available anytime and anywhere (100). This has been the main response in reforming dental education during outbreaks of respiratory infectious diseases. Beyond technical efficacy, the successful implementation of AI and VR in dental education depends heavily on student acceptance. According to the Technology Acceptance Model (TAM), perceived usefulness (e.g., whether AI can rapidly deliver accurate information support) and perceived ease of use (e.g., user interface and feedback responsiveness) are critical determinants of students' behavioral intention to adopt these tools (101). Recently, a number of researchers have started employing the TAM to assess the perceptions of both faculty and students concerning the integration of these novel technologies into teaching reform (102, 103). Therefore, future educational strategies should prioritize optimizing the user experience of these AI and VR assistants for information support to enhance their adoption among dental learners.

Based on TAM, our review identifies several strategic policies for curriculum design that can improve student learning effectiveness during and beyond respiratory disease outbreaks. First, perceived usefulness can be enhanced by explicitly linking digital tools to clearly defined learning outcomes. For classroom teaching, curricula should integrate AI-augmented PBL/CBL that demonstrates tangible benefits—such as faster access to relevant clinical information, real-time feedback, and exposure to a broader range of clinical scenarios than traditional methods allow. For laboratory and clinical teaching, AR/VR systems should be introduced with clear competency-based objectives, showing students how simulation directly translates into improved clinical skills and patient safety. Second, perceived ease of use can be improved through user-centred design and adequate training. Curricula should include structured onboarding modules that familiarise students with digital platforms, ensuring that technical complexity does not become a barrier to adoption. Institutions should also provide technical support hotlines, clear user manuals, and peer mentoring schemes to reduce frustration and increase confidence. Third, institutional policies should align with TAM by fostering a culture of innovation through faculty development programmes, recognition of teaching-technology integration, and investment in reliable infrastructure (stable networks, updated hardware, and accessible platforms). Fourth, assessment and feedback mechanisms should be redesigned to incorporate digital tools in ways that students perceive as fair, transparent, and valuable—for example, using AI for formative, low-stakes feedback before high-stakes summative assessments, thereby building trust and perceived usefulness.

The COVID-19 pandemic and recurrent outbreaks of other respiratory infectious diseases have posed unprecedented challenges to dental education systems worldwide. In China, dental schools were compelled to rapidly overhaul their pedagogical approaches, clinical training modalities, student admission procedures, and licensing examinations, all while ensuring the safety of both students and patients. Drawing on the collective experience of Chinese dental institutions over the past several years, a range of innovative strategies have been piloted—from online classrooms and virtual simulation laboratories to AI-assisted assessments and remote proctoring. Yet, these adaptations have also revealed persistent barriers, including infrastructure limitations, digital inequality, faculty development gaps, and unresolved questions about the validity and fairness of virtual evaluations. The table below (Table 1) summarises thematic synthesis of findings from across five core domains—classroom teaching, laboratory instruction, clinical education, student admissions and assessment, and licensing examinations—outlining the main solutions that have been applied and the critical challenges that remain. This overview is intended to inform educators, administrators, and policymakers both within China and internationally, as they seek to build more resilient and effective dental education systems for future epidemic scenarios.

**Table 1:** Thematic synthesis of findings from the narrative review with content analysis: solutions, educator challenges, and student challenges across five domains of dental education.

Aspect	Potential solutions	Challenges for educators	Challenges for students
Classroom Teaching	Integrate dental public health and infectious disease content into the curriculum (15, 16) Adopt online teaching (17–20) Implement MOOCs, SPOCs, flipped classrooms (21–23) Use AI to enhance PBL/CBL with multimodal cases and personalised feedback (31–33)	Network instability and hardware/software limitations (25) Reduced teacher-student interaction and engagement (25, 26) Need for information-based teaching capabilities (30) AI barriers: cost, bias, transparency, ethical concerns, resistance to change (34)	Heavy reliance on self-discipline and motivation for learning Digital inequities: rural and low-socioeconomic students face severe disadvantages in access to technology and network quality (27, 28) Insufficient learning atmosphere and reduced peer interaction (25)
Laboratory Teaching	Use remote teaching for independent tasks Employ AR/VR simulation systems (38–44) Utilise national sharing platforms (iLab-X) for online lab modules (52)	High equipment cost; limited units cannot serve large cohorts Haptic feedback and user-interface realism still inadequate (50) Inability to simulate patient emotions/physiological responses (51) Need for improvement in simulation realism, interaction design, and data analytics (53)	Inability to practice at home due to dependence on specialised, expensive VR equipment Lack of authentic tactile feedback compared to natural teeth
Clinical Teaching	Staggered schedules and spaced-out dental chairs (64) Mandatory PPE (masks, face shields, goggles, gowns) (68) Use rubber dams, high-volume suction, pre-procedural mouthwash (69) Apply negative-pressure rooms and HEPA air filtration (70, 71)	Heavy burden of enforcing rigorous infection control protocols, environmental disinfection, and ventilation Reduced patient flow and clinical schedules due to social distancing (60, 61) Undergraduate interns perform slower than other trainees, restricting clinical teaching capacity (59) Negative-pressure rooms are rarely available in most dental clinics (70, 71)	Reduced patient exposure and limited clinical practice opportunities, potentially compromising skill acquisition (59, 61) Bio-aerosol generation poses an infection risk during training (55, 56) Anxiety about personal safety and family health when attending in-person clinical sessions
Student Admissions and Assessment	Use online exams and interviews with dual-camera proctoring (73) Replace standard-answer questions with open-ended items Implement virtual OSCEs and AI-generated examinations (78, 81, 83) Use VR/AR for practical skills assessment with digital scoring (86) Apply AI for item generation, grading, and formative feedback (84) Develop hybrid (online/onsite) assessment models	Lack of remote technical measures to prevent cheating (74) Subjective scoring and validity concerns for open-ended questions (74) AI concerns: accuracy, bias, ethical implications (85) Need to establish new, appropriate evaluation criteria for diverse online formats	High dependence on stable network and personal IT equipment; technical glitches cause stress (74) Concerns that practical skills are undervalued compared to theoretical knowledge Worries about algorithmic bias and lack of transparency in AI scoring (85) Skepticism about whether virtual OSCEs truly reflect real clinical competence (80) Disrupted internships and clinical exposure hamper professional development (76)
Licensing Examinations	Schedule centralised in-person written exams at safe time windows with strict protection protocols (88) Explore VSP for clinical skills testing (91–93) Investigate AI for objective scoring and item generation (89, 90) Develop pre-approved contingency protocols for outbreaks	National exams cannot easily alter format/content/validity Dual-camera proctoring and open-ended questions not feasible Clinical skills exams lack satisfactory alternative VSP high cost and limited validation across competencies (93) LLM research mainly on question generation, not scoring (90) Concerns about patient safety if exams are postponed/cancelled	Geographic limitations force long-distance travel, incurring high costs and infection exposure risk Delays/postponements cause career disruptions, financial dependence, and heightened anxiety Cautious about AI for evaluation due to fear of opaque algorithms and bias Demand for assessment methods that are fair, transparent, and accurately measure clinical readiness

A narrative review is a traditional review that describes the literatures (13), which primarily aims to collect empirical and theoretical evidence related to a problem, issue, or topic, with a focus on known subject content and the methodologies, models, and concepts that others have applied to the topic. After defining the topic, it provides a search strategy; it adopts an unstructured approach to data extraction, typically without using a data extraction form. Finally, it examines and synthesises the literature in a narrative and/or tabular format, incorporating the author's critical interpretation of the evidence (104, 105). Nevertheless, its shared hallmark is the examination of published work, with the aim of identifying prior accomplishments in order to consolidate, build upon, summarise, avoid redundancy, and detect omissions or gaps in the existing literature. However, any conclusions derived from such reviews may be susceptible to bias, may overlook significant portions of the literature, or may not critically interrogate the validity of the claims presented. Moreover, authors might selectively include only those studies that align with their own perspectives, thereby disproportionately reinforcing preferred assumptions (106). Highquality narrative reviews are both feasible and desirable, and they will continue to serve an important function in medicine (107). Nevertheless, several limitations of the present narrative review merit careful consideration. First, given its narrative rather than systematic nature, the review was inherently subject to potential selection bias, as the literature search, although structured, was not exhaustive and may have inadvertently omitted relevant studies published in databases not consulted or in the gray literature. Second, the overwhelming majority of identified literature pertained to the COVID-19 pandemic, which inevitably skewed the discussion toward SARS-CoV-2, whereas evidence regarding other respiratory pathogens—such as influenza, tuberculosis, or emerging airborne viruses—remains comparatively sparse, limiting the generalizability of our findings across the full spectrum of respiratory infectious diseases. Third, the review placed a deliberate emphasis on the Chinese dental education context, reflecting the authors' regional expertise and accessibility to local institutional data; however, this geographical focus may restrict the transferability of the conclusions to other socio-cultural and healthcare systems with different epidemiological profiles and educational infrastructures. Fourth, the considerable heterogeneity among respiratory diseases—in terms of transmission dynamics, infection control requirements, and clinical severity—poses challenges in synthesizing uniform recommendations, and caution should be exercised when extrapolating pandemic-specific adaptations to endemic respiratory infections. Finally, the rapid pace of technological innovation in simulation-based training, telehealth, and infection control equipment, coupled with the frequent updates to public health guidelines, means that certain findings and recommendations discussed herein may become outdated swiftly. Therefore, our interpretations should be viewed as a contemporary snapshot rather than a definitive or permanent synthesis.

## 5 Conclusions

The field of dental education urgently requires a comprehensive set of countermeasures to combat potential future epidemics and outbreaks of various respiratory infectious diseases. China's experience during respiratory epidemics underscores both the remarkable adaptability and the persistent fragility of conventional dental education systems. Our review reveals that while rapid pivots to online platforms, AR/VR simulation, enhanced infection control, and remote assessments succeeded in maintaining educational continuity, these measures have produced two distinct but interconnected sets of implications that are critical for both curriculum design and student outcomes.

For curriculum design and institutional planning, the findings indicate that dental schools must move beyond ad-hoc crisis responses toward structured, evidence-based curricula that embed respiratory infectious disease preparedness across all training stages. This includes integrating public health and infection control content into classroom teaching, allocating sustainable funding for high-cost simulation infrastructure, redesigning clinical rotations to balance patient safety with skill acquisition, and developing flexible admissions and licensing protocols that can be activated during outbreaks. The digital divide, equity issues, and faculty development needs identified in this review should be addressed through targeted policy interventions at both institutional and national levels.

For student learning outcomes, the evidence demonstrates that while online and simulation-based modalities can effectively deliver theoretical knowledge and basic technical skills, they have not yet proven fully comparable to traditional hands-on clinical experience in developing nuanced clinical judgment, patient communication, and psychomotor proficiency. Students' self-discipline, digital literacy, and access to reliable technology significantly influence their performance, raising concerns about fairness and the validity of remote assessments. Moreover, reduced patient exposure during outbreaks risks compromising graduates' clinical readiness, which has direct implications for patient safety and professional confidence.

Looking forward, future preparedness must prioritise longitudinal research to validate the transfer of skills acquired through simulation and online training to real-world clinical practice. AI-driven tools and immersive technologies, though promising, require careful evaluation for accuracy, bias, cost-effectiveness, and ethical acceptability before they can be integrated as formal components of curricula and high-stakes assessments. Ultimately, building resilient, inclusive, and flexible educational frameworks—anchored in clear curriculum objectives and measurable student outcomes—is essential to safeguard both training quality and patient safety, and to ensure that the dental workforce remains competent and confident in the face of future respiratory infectious disease outbreaks.
